# Reference miRNAs for miRNAome Analysis of Urothelial Carcinomas

**DOI:** 10.1371/journal.pone.0039309

**Published:** 2012-06-20

**Authors:** Nadine Ratert, Hellmuth-Alexander Meyer, Monika Jung, Hans-Joachim Mollenkopf, Ina Wagner, Kurt Miller, Ergin Kilic, Andreas Erbersdobler, Steffen Weikert, Klaus Jung

**Affiliations:** 1 Department of Urology, University Hospital Charité, Berlin, Germany; 2 Berlin Institute for Urologic Research, Berlin, Germany; 3 Institute of Physiology, University Hospital Charité, Berlin, Germany; 4 Max Planck Institute for Infection Biology, Berlin, Germany; 5 Institute of Pathology, University Hospital Charité, Berlin, Germany; 6 Institute of Pathology, University Rostock, Rostock, Germany; The University of Arizona, United States of America

## Abstract

**Background/Objective:**

Reverse transcription quantitative real-time PCR (RT-qPCR) is widely used in microRNA (miRNA) expression studies on cancer. To compensate for the analytical variability produced by the multiple steps of the method, relative quantification of the measured miRNAs is required, which is based on normalization to endogenous reference genes. No study has been performed so far on reference miRNAs for normalization of miRNA expression in urothelial carcinoma. The aim of this study was to identify suitable reference miRNAs for miRNA expression studies by RT-qPCR in urothelial carcinoma.

**Methods:**

Candidate reference miRNAs were selected from 24 urothelial carcinoma and normal bladder tissue samples by miRNA microarrays. The usefulness of these candidate reference miRNAs together with the commonly for normalization purposes used small nuclear RNAs RNU6B, RNU48, and Z30 were thereafter validated by RT-qPCR in 58 tissue samples and analyzed by the algorithms geNorm, NormFinder, and BestKeeper.

**Principal Findings:**

Based on the miRNA microarray data, a total of 16 miRNAs were identified as putative reference genes. After validation by RT-qPCR, miR-101, miR-125a-5p, miR-148b, miR-151-5p, miR-181a, miR-181b, miR-29c, miR-324-3p, miR-424, miR-874, RNU6B, RNU48, and Z30 were used for geNorm, NormFinder, and BestKeeper analyses that gave different combinations of recommended reference genes for normalization.

**Conclusions:**

The present study provided the first systematic analysis for identifying suitable reference miRNAs for miRNA expression studies of urothelial carcinoma by RT-qPCR. Different combinations of reference genes resulted in reliable expression data for both strongly and less strongly altered miRNAs. Notably, RNU6B, which is the most frequently used reference gene for miRNA studies, gave inaccurate normalization. The combination of four (miR-101, miR-125a-5p, miR-148b, and miR-151-5p) or three (miR-148b, miR-181b, and miR-874,) reference miRNAs is recommended for normalization.

## Introduction

MicroRNAs (miRNAs) belong to a class of small noncoding RNAs of 19 to 24 nucleotides that are known to regulate signaling pathways for various cell functions. Not surprisingly, changes in miRNA expression have been associated with several diseases, including cancer [1], [2]. It has been shown that different tumors have specific miRNA expression profiles and that miRNA profiles correlate with patient diagnosis, prognosis, and responses to treatment [3]. Thus, analyzing the differential expression of the microRNAome [4], defined as the entirety of all miRNAs in a cell, is of scientific and practical significance.

Several methods such as bead-based flow cytometry, microarray, deep sequencing, and real-time quantitative PCR (RT-qPCR) allow fast, high-throughput, and sensitive profiling of miRNAs. RT-qPCR produces specific, sensitive, and reproducible quantification of nucleic acids. To overcome experimental variations in RT-qPCR analyses (RNA isolation, cDNA synthesis, PCR runs), relative quantification of miRNAs of interest based on the normalization to reference genes is the approach of choice to prevent errors within a dataset [5]. This approach complies with normalization procedures used in mRNA expression studies and is summarized in the recent MIQE guidelines [6]. Suitable reference genes should be expressed constitutively and be independent of biological changes, diseases or treatments. The use of multiple rather than single reference genes has been recommended for RT-qPCR data normalization [7], [8]. The computional programs geNorm [9] and NormFinder [10] are based on this principle. These tools allow identifying the most stable reference genes from a panel of putative reference genes for normalization. Moreover, several studies in addition to our own experiments have shown that the use of inappropriate reference genes in the relative quantification of gene expression can result in biased expression profiles [11]–[13]. As there are no universal reference genes [14], [15], it is strongly recommended that researchers test for the most suitable reference genes specific to the tissues and experimental conditions used.

Because of our general interest on miRNAomes in urological tumors and the increasing incidence of urothelial cancer [16], we performed a literature search in PubMed. The MeSH term “microRNAs” was combined with the search string [“microRNAs” OR “microRNA” OR “miRNA” OR “miRNAs”] and in combination with the MeSH term “urinary bladder neoplasms”. Fifty-eight articles published until May 2012 were identified, of which 27 investigated miRNA expression. Specifically, 20 publications reported miRNA expression by RT-qPCR and used small nuclear, nucleolar or ribosomal RNAs as well as mRNAs for normalization, namely RNU6B (15 times) [17]–[31], RNU48 (6 times) [17], [18], [32]–[35], RNU43 (1 time) [30], RNU44 (1 time) [18], beta-actin (1 time) [36], and 18srRNA (1 time) [32] without confirming their validity for normalization. Thus, no systematic study has been performed to identify suitable miRNA reference genes in urothelial carcinoma while corresponding studies have been performed for other cancer entities, including urological tumors [13], [37]–[40].

Bladder cancer is the fourth most common cancer in Western industrialized countries [16]. Approximately 90% of all urothelial neoplasms are classified as urothelial carcinoma. Although surgical techniques and treatments have improved over time, bladder cancer is still a common cancer with a high mortality. To date, mechanisms of urothelial carcinogenesis have not been fully elucidated. However, miRNA expression patterns have been linked to clinical outcomes in urothelial carcinoma [18], [24]. Therefore, single miRNA biomarkers or biomarker signatures of multiple miRNAs may improve risk stratification of patients and may supplement the histological diagnosis of urological tumors including bladder cancer [24], [41]–[43]. In addition, miRNAs and their regulated genes represent interesting drug targets because miRNAs can influence the expression of multiple genes and thereby affect numerous points in disease pathways [22], [44]–[46]. The significance of miRNAs in the regulation of signal transduction in bladder cancer was recently summarized [47]. Improved knowledge in this field will contribute to enhanced prognosis and selection of treatment strategies. However, as mentioned above, accurate quantification of miRNA expression by RT-qPCR and thus reliable expression data require proper normalization strategies. Computer programs based on various algorithms including geNorm [9], NormFinder [10], and BestKeeper [48] have been developed to rank putative reference genes according to their expression stability and indicate the best reference gene or combination of reference genes for accurate normalization.

In the present study, we aimed to systematically identify suitable reference genes for normalizing RT-qPCR assays of miRNA expression in urothelial carcinoma tissue. Using miRNA microarray analyses, we first identified invariant miRNAs that showed stable expression in both nonmalignant and malignant bladder tissue samples as candidate reference miRNAs. RT-qPCR analyses were subsequently performed for validating these miRNAs from the microarray experiments and the above mentioned small RNAs RNU6B, RNU48, and Z30 as putative reference genes. Appropriate reference miRNAs were identified by geNorm, NormFinder, and BestKeeper, and the results of unsuitable normalization are illustrated with invalid normalizers.

## Materials and Methods

### Patients and Tissue Samples

All bladder cancer patients went through radical cystectomy or transurethral resection at the University Hospital Charité in Berlin between 2008 and 2009 and gave written informed consent for the use of representative tissue specimens for research purposes. The study was approved by the Ethic Committee of the University Hospital Charité (File: EA1/153/07). The samples were collected immediately after surgery in liquid nitrogen and stored at −80°C until further analysis. Tumor staging was performed in conformity with the International Union Against Cancer [49] and histological grading in accordance with the WHO/ISUP criteria of 2004 [50]. In total, 58 urothelial samples were included in this study. Seventeen samples were from nonmalignant bladder tissue (15 male, 2 female patients; median age 68, range 47–80 years), 20 samples were from low-grade papillary urothelial carcinoma (18 male, 2 female patients; median age 68, range 50–86 years), and 21 samples were from high-grade tumors (14 male, 7 female patients; median age 73, range 48–82 years).

### Isolation of RNA and Characterization of Quantity and Quality

Frozen histologic sections were prepared, stained with hematoxylin/eosin, and examined by uropathologists (A.E., E.K.). Only tissue specimens with more than 80% tumor cells were included in the study as tumor samples. Tissue cryotome sections (approximately 20–30 mg of tissue, wet weight) were treated with 350 µl of lysis buffer and total RNA including miRNAs was isolated using the miRNeasy Mini Kit (Qiagen, Hilden, Germany) with 30 to 50 µl of elution buffer according to the manufacturer’s protocol. An additional DNase I digestion step on the RNA binding silica gel membrane of the spin column was performed. RNA concentration and the 260 nm to 280 nm absorbance ratios were measured on the NanoDrop 1000 spectrophotometer (NanoDrop Technologies, Wilmington, DE, USA). The quality of isolated RNA was determined by the RNA integrity number (RIN) with a Bioanalyzer 2100 (Agilent Technologies, Santa Clara, CA, USA). Only samples with RIN values >5 were used. The RNA samples (medians: 693 ng/µl; 830 ng/mg tissue) isolated from nonmalignant as well as from low-grade and high-grade tumor tissue samples showed comparable median 260/280 absorbance ratios (2.02, 2.03, and 2.03) and RIN values (6.7, 5.9, and 6.3; Kruskal-Wallis test, P = 0.171).

### Microarray-based miRNA Analysis

Microarray analyses of eight samples each from nonmalignant tissue and low and high grade tumor specimens were performed. One-color hybridizations on human catalog 8-plex 15 K microRNA microarrays (AMADID 019118) from Agilent encoding probes for 723 human and 76 viral microRNAs from the Sanger database v10.1 were used. All reaction steps were carried out as previously described in detail [51]. After hybridization, microarrays were washed, scanned, and processed according to the supplier’s protocol. The raw data were normalized using Genespring GX11 Software (Agilent) with default parameters (threshold raw signal to 1.0, percent shift to 90th percentile as normalization algorithm and no baseline transformation). All microarray data have been deposited in the NCBI GEO database with accession number GSE36121. Further data analysis is described in the Results section.

### Quantitative Real-time PCR

RT-qPCR analyses of miRNAs were carried out with TaqMan microRNA assays (Applied Biosystems, Foster City, CA, USA) according to the manufacturer’s protocol and the MIQE guidelines [6] (Table S1) as previously described [13]. The reverse transcription of miRNAs from total RNA (10 ng) was performed with miRNA-specific stem-loop primers, 10 nmol dNTP mix, 2.6 U of RNase Inhibitor, 33.5 U of MultiScribe RT enzyme, and 1 × RT Buffer (Applied Biosystems). The generated cDNAs were stored at 20°C until analysis. The qPCR measurements were executed in white 96-well PCR plates (cat.no. 04729692001 with sealing foils) with a 10 µl of final volume containing 1 µl of RNA-specific cDNA, 1× TaqMan Universal PCR Master Mix No AmpErase UNG, and gene-specific TaqMan MiRNA real-time PCR assay solution on the Light Cycler 480 Instrument (Roche Diagnostics GmbH, Mannheim, Germany; software version 1.5.0) (Table S2). The reaction was performed at 95°C for 10 min, followed by 45 cycles of 95°C for 15 s, and 60°C for 60 s. All samples were measured in triplicate; each PCR run included a no-template control and two inter-plate calibrators. All no-template controls were negative. To assess the specific amplification efficiencies, we created calibration curves from dilution series of miRNA-specific cDNAs or small nuclear RNAs (Methods S1). The efficiency was determined from the slope of the log regression plot of Cq values versus log input of cDNA. Efficiencies were in the range between 81% and 88%. All data were corrected to the PCR efficiency and to the inter-run calibrators. For that purpose, the software qBase^PLUS^ (Biogazelle NV, Zwijnaarde, Belgium) was used, employing a generalized and universally applicable quantification model based on efficiency correction, error propagation, and multiple reference gene normalization [52]. The intra-run precision for the finally considered candidate reference miRNAs miR-29c, miR-101, miR-125a-5p, miR-148b, miR-151-3p, miR-151-5p, miR-181a, miR-181b, miR-324-3p, miR-424, and miR-874 as well as the investigated small nuclear RNU6B, RNU48, and Z30 ranged from 0.15% to 0.35% for mean Cq values between 21.93 to 26.65. The between-run precision (n = 42) measured for the control miR-21 was found to be 1.62% (mean Cq ± standard deviation: 28.35±0.46).

### Data Analysis

Statistical analyses were performed using GraphPad Prism Version 5.04 (GraphPad Software Inc., La Jolla, CA, USA). Non-parametric tests (Mann-Whitney U test; Kruskal-Wallis test with Dunn’s multiple comparison test) were used to analyze significant differences between independent groups. The Spearman correlation coefficients were applied to calculate the relationships between the miRNAs as well as between the clinical variables and the expression of candidate reference miRNAs. P values <0.05 (two-tailed) were considered statistically significant.

The assessment of the putative reference genes for normalization was evaluated by the computer programs geNorm [9] using the improved version geNorm^Plus^ as an implementation of the software qBase^PLUS^ (Biogazelle, Belgium) [52], NormFinder [10], and BestKeeper [48].

## Results

### Selection of Candidate Reference miRNAs by Microarray Analysis

To identify putative reference miRNAs in the miRNA microarray data obtained from the eight samples of each tissue group, the following criteria were used: (a) miRNAs had to be detected in Genespring GX11 software as “present” in all examined 24 samples to filter out signals that did not reach a minimum of intensity, (b) the absolute fold change between the nonmalignant and the two cancerous groups had to be lower than 1.2-times with (c) no significant differences (P>0.05) between the groups. Based on the total of 723 human miRNA species located on the Agilent microarray chip according to the miRBase version 10.1, we identified 101 miRNAs that were flagged as “present” in all of the examined groups (Table S3). Eight of these miRNAs showed absolute fold changes lower than 1.2-times and had no significant differences between the groups (Table 1, indicated by the symbol “N”). To avoid normalization artifacts of the microarray data, we also used raw microarray expression data. Thus, with the criteria mentioned above, we revealed a second set of eight candidate reference miRNAs (Table 1, indicated by symbol “R”). Taking these sets together, 16 putative reference miRNAs were included in further analyses (Table 1; Table S2).

**Table 1:** Candidate reference miRNAs selected from microarray analysis.†

miRNA&	ID according miRBase version#	Selection criterion§
*hsa-miR-15a*	hsa-miR-15a (v10.1)	R
	hsa-miR-15a-5p (v18)	
*hsa-miR-20b*	hsa-miR-20b (v10.1)	R
	hsa-miR-20b-5p (v18)	
hsa-miR-29c	hsa-miR-29c (v10.1)	R
	hsa-miR-29c-3p(v18)	
hsa-miR-101	hsa-miR-101 (v10.1)	N
	hsa-miR-101-3p (v18)	
*hsa-miR-107*	hsa-miR-107 (v10.1)	N
	hsa-miR-107 (v18)	
hsa-miR-125a-5p	hsa-miR-125a-5p (v10.1)	N
	hsa-miR-125a-5p (v18)	
hsa-miR-148b	hsa-miR-148b (v10.1)	R
	hsa-miR-148b-3p (v18)	
hsa-miR-151-3p	hsa-miR-151-3p (v10.1)	R
	hsa-miR-151-3p (v18)	
hsa-miR-151-5p	hsa-miR-151-5p (v10.1)	N
	hsa-miR-151-5p (v18)	
hsa-miR-181a	hsa-miR-181a (v10.1)	R
	hsa-miR-181a-5p (v18)	
hsa-miR-181b	hsa-miR-181b (v10.1)	R
	hsa-miR-181b-5p (v18)	
hsa-miR-324-3p	hsa-miR-324-3p (v10.1)	N
	hsa-miR-324-3p (v18)	
hsa-miR-424	hsa-miR-424 (v10.1)	N
	hsa-miR-424-5p (v18)	
*hsa-miR-513a-5p*	hsa-miR-513a-5p (v10.1)	R
	hsa-miR-513a-5p (v18)	
hsa-miR-874	hsa-miR-874 (v10.1)	N
	hsa-miR-874 (v18)	
*hsa-miR-939*	hsa-miR-939 (v10.1)	N
	hsa-miR-939 (v18)	

† The TaqMan MicroRNA Assay ID, miRBase accession number, and the sequence for each miRNA are compiled in Table S2.

& miRNAs marked in Italics were not included in further analyses because their low expression level was beyond the dynamic range of the assay (>35Cq) (further details see text).

# The miRNA ID from the miRBase version 10.1 and 18, respectively.

§ Symbols “N” and “R” indicate the selection of the candidate reference miRNAs based on normalized or raw microarray data as described in the text.

### Validation of Candidate Reference Genes by RT-qPCR

To increase the statistical power to find suitable reference miRNAs, in addition to the 24 analyzed samples in the microarray experiments, we included nine nonmalignant and 25 malignant tissue samples as described in the section “Patients and tissue samples” to validate the aforementioned 16 candidate reference miRNAs in more detail by RT-qPCR. Furthermore, the set of candidate reference miRNAs was extended by the small RNAs RNU6B, RNU48, and Z30 that were commonly used for expression normalization in the literature as stated in the Introduction. First, to determine if reliable quantification of these putative normalizers is feasible by RT-qPCR, three RNA pools were prepared containing equal amounts of RNA from the samples used in the microarray analysis. miR-15a, miR-20b, miR-107, miR-513a-5p, and miR-939 showed Cq values >35 in the pools and were excluded from further analyses because accurate quantification would be questionable. By this preselection, 11 putative reference miRNAs (Table 1: miR-29c, miR-101, miR-125a-5p, miR-148b, miR-151-3p, miR-151-5p, miR-181a, miR-181b, miR-324-3p, miR-424, and miR-874) as well as RNU6B, RNU48, and Z30 were further investigated and showed Cq values ranging from 22 (RNU48) to 28 (miR-324-3p).

**Figure 1:**
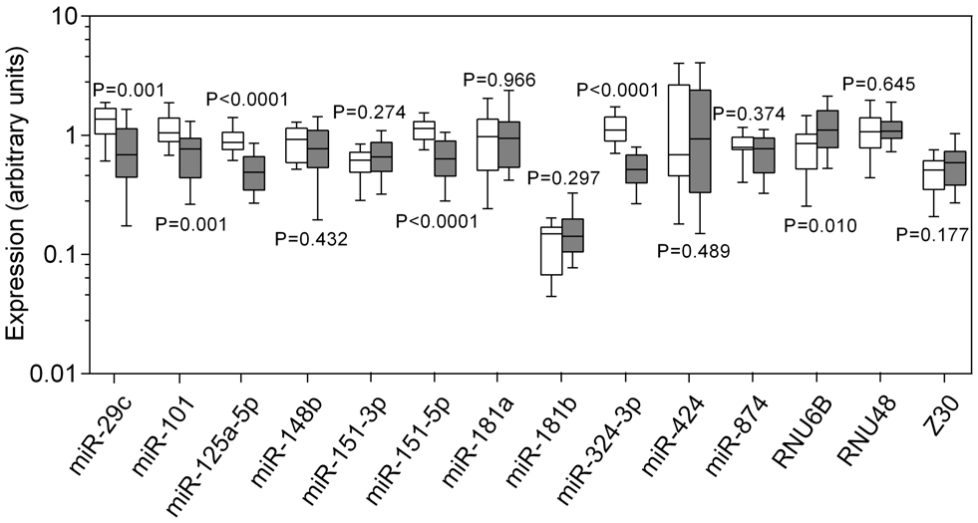
Expression of candidate reference genes in human nonmalignant and malignant bladder tissue samples. RT-qPCR analyses were performed from 17 nonmalignant bladder tissue samples and 41 samples from low-grade and high-grade papillary urothelial carcinoma. Expression levels of the candidate reference genes are given as arbitrary units. Boxes (blank, nonmalignant samples; black, malignant samples) represent lower and upper quartiles with median as horizontal line; whiskers depict the 10 and 90 percentiles. Significances are illustrated as *P* values of the Mann-Whitney *U* test.

In the second step, all 14 reference candidates were separately measured in the 58 samples (Figure 1). The expression levels significantly differed for miR-29c (P = 0.0012), miR-101 (P = 0.0007), miR-125a-5p (P<0.0001), miR-151-5p (P<0.0001), miR-324-3p (P<0.0001), and RNU6B (P = 0.0101) between nonmalignant and malignant samples. The remaining eight miRNAs, namely miR-148b, miR-151-3p, miR-181a, miR-181b, miR-424, miR-874, RNU48, and Z30, revealed no significant differences between nonmalignant and malignant samples (P>0.05). We followed the general recommendation of the geNorm program and included all these putative reference miRNAs and small RNAs in further analyses for reassessing their potential contribution as normalizers. However, miR-151-3p was excluded due to the fact that miR-151-3p and miR-151-5p are mature miRNAs of the same pre-miRNA and miR-151-5p exhibited higher expression in examined samples.

### Association between the Candidate Reference miRNAs and Clinical Variables

The correlation between the putative reference miRNAs and the correlation of these miRNAs with age, sex, and tumor characteristics were determined. Spearman rank correlations are summarized in Table S4. Classifying miRNA pairs with coefficients ≥0.60 as co-expressed, we identified this characteristic co-expression feature among the four miRNAs miR-101, miR-125a-5p, miR-151-5p and miR-324-3p as well as between miR-148 and miR-151-3p, and between miR-181a and miR-181b. The correlation between miR-101, miR-151-5p, and miR-324-3p was remarkable.

The expression of the 11 miRNAs and three small RNAs was not associated with age (Spearman rank correlation from r_S_ −0.23 to 0.177, P values from 0.083 to 0.974), sex (Mann-Whitney U test, P values from 0.062 to 0.851), or tumor stage (Ta, T1, T2, T3; Kruskal-Wallis test, P values from 0.092 to 0.826, except for miR-29c, which had P = 0.044). Differences in expression between low-grade and high-grade tumors were found for miR-29c (down-regulated, P = 0.005), miR-874 (down-regulated, P = 0.019), miR-181a (up-regulated, P = 0.031), and miR-181b (up-regulated, P = 0.0008), while all other miRNAs were not differentially expressed (P values from 0.092 to 0.826).

### Identification of Suitable Reference miRNAs using geNorm, NormFinder, and BestKeeper

**Figure 2:**
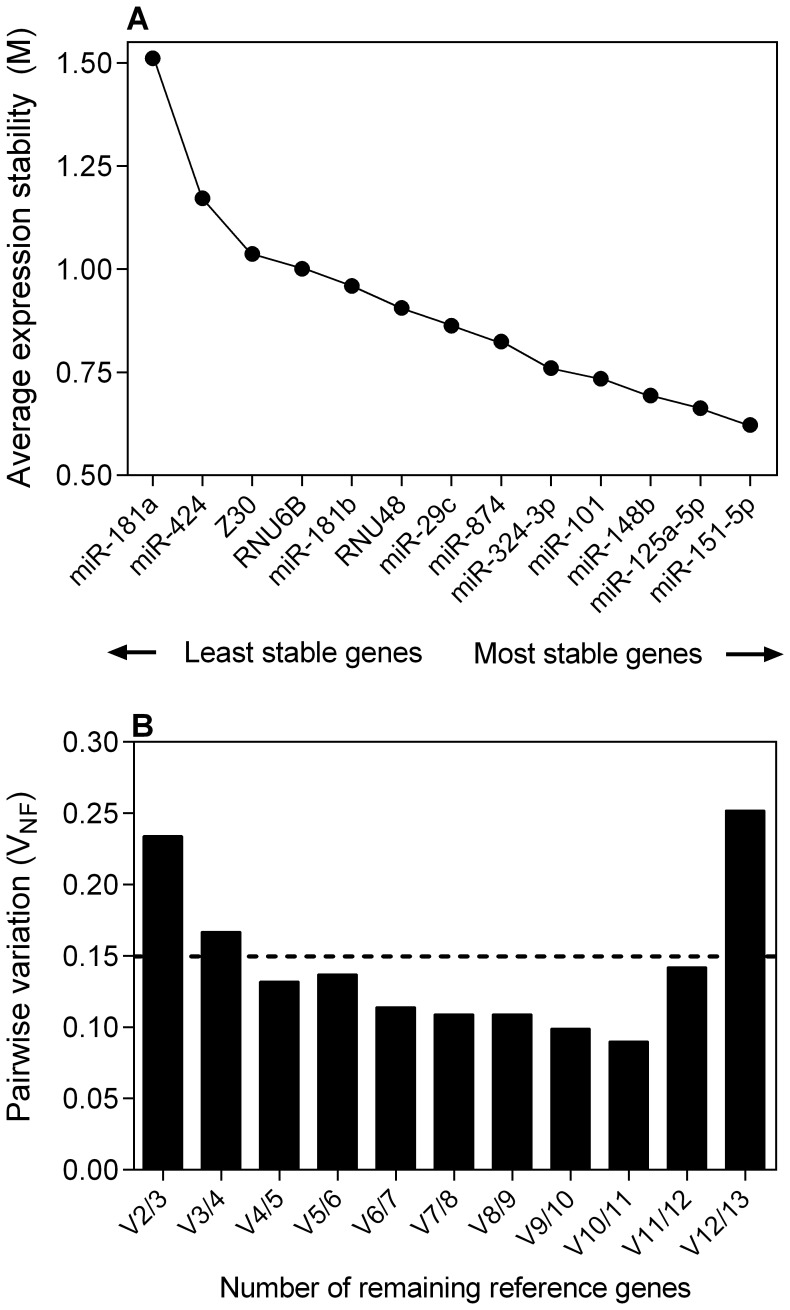
geNorm analysis of RT-qPCR-based candidate reference genes. (A) The geNorm analysis shows the calculation of the average expression stability value M of all candidate reference genes determined by RT-qPCR. Genes with the highest M value have the least stable expression, while the genes with the lowest M value have the most stable expression. The x-axis presents the ranking of reference genes in order of increasing stability from left to the right. (B) Calculation of the optimal number of reference genes for normalization. geNorm calculates a normalization factor assessing the optimal number of reference genes for generating that factor. The normalization factor is calculated from at least two genes taking into account the variable V as the average pairwise variation (V_NF_) between two sequential normalization factors. The thin broken line illustrates the cut-off value V_NF_ <0.15. In this experiment, the optimal number of reference genes was four (V4/5). geNorm shows the variation of the normalization factor of four genes as the best combination (miR-101, miR-148b; miR-125a-5p, and miR-151-5p) in relation to five genes as shown in (A) and in the following order. All the results are presented according to the output files of the geNorm program.

To identify suitable reference genes for the normalization of miRNA expression, we applied the aforementioned three computer programs geNorm, NormFinder, and BestKeeper. GeNorm, an implementation of the new software qBase^PLUS^, automatically returns the average expression stability value M and the average pairwise variation V of a particular gene with all other control genes. The highest M value indicates the gene with the least stable expression. Figure 2A and Table 2 indicate the M values and the resulting ranking order of all investigated candidate reference miRNAs and small RNAs based on expression stability. miR-181a (M = 1.511) showed the highest M value, whereas miR-151-5p (M = 0.622) and miR-125a-5p (M = 0.663) showed the lowest M values. Consequently, miR-181a had the least stable expression while miR-125a-5p and miR-151-5p had the most stable expression. Additionally, geNorm calculates a normalization factor (V_NF_ value), which is a criterion for the optimum number of reference genes (Figure 2B). The program recommends V_NF_ values less than 0.15 for proper normalization. When this cut-off value is achieved, it is not necessary to include any additional reference genes. As illustrated in Figure 2B, the four miRNAs miR-101, miR-148b, miR-125a-5p, and miR-151-5p (V_NF_ value 0.14) were recommended as an optimum reference miRNA set for normalization; the best combination of two reference miRNAs was miR-125a-5p and miR-151-5p, and the best single reference miRNA was miR-151-5p. After excluding the potentially deregulated reference miRNAs mentioned above, geNorm analysis was repeated. However, under these conditions, geNorm calculated a V_NF_ value >0.15 and therefore did not recommend a normalization set.

**Table 2:** Ranking of candidate reference miRNAs and small RNAs in human nonmalignant and malignant bladder tissues according to their stability value using geNorm, NormFinder, and BestKeeper algorithms.

	geNorm		NormFinder		BestKeeper	
Gene name	Stability value&	Rank	Stability value&	Rank	SD [±x-fold]#	Rank
**miR-101**	0.734	4	0.215	8	0.69	10
**miR-125a-5p**	0.663	2	0.192	6	0.62	5
**miR-148b**	0.693	3	0.086	1	0.65	8
**miR-151-5p**	0.622	1	0.230	9	0.60	3
**miR-181a**	1.511	13	0.209	7	–	
**miR-181b**	0.959	9	0.155	3	0.62	6
**miR-29c**	0.863	7	0.246	10	–	
**miR-324-3p**	0.76	5	0.291	11	0.64	7
**miR-424**	1.172	12	0.371	13	–	
**miR-874**	0.824	6	0.102	2	0.53	2
**RNU6B**	1.001	10	0.349	12	0.67	9
**RNU48**	0.906	8	0.173	5	0.51	1
**Z30**	1.037	11	0.171	4	0.61	4
**Best gene**	miR-151-5p		miR-148b		RNU48	
**Best combination**	miR-101, miR-125a-5p, miR-148b, miR-151-5p		Z30, miR-125a-5p		–	

& High expression stability is indicated by low stability value.

# SD [±x-fold]: standard deviation of the absolute regulation coefficients. SD >1 can be considered inconsistent.

Similar to geNorm, NormFinder identified genes with the lowest M values as the most stably expressed reference targets (Table 2). NormFinder ranked the four best reference genes for normalization as miR-148, miR-874, miR-181b, and Z30. Z30 and miR-125a-5p were recommended as the best combination, and miR-148b was recommended as the best single normalizer (Table 2).

BestKeeper considers all genes in all observed groups. First, BestKeeper determines the geometric mean and coefficient of variance. Genes with a standard deviation greater than 1 were assumed to be inconsistent. In the second step, the inter-gene relationships were examined by pairwise correlation analysis. This calculation is used to determine whether the gene expression exhibits a similar behavior. Candidate reference genes that highly correlate with each other are included in the BestKeeper-Index calculation. The program provides only an analysis of ten candidate reference genes simultaneously. Therefore, we excluded the reference targets with the lowest M values as determined by geNorm (miR-181a) and NormFinder (miR-424) and also excluded miR-29c (rank 9 by geNorm; rank 11 by NormFinder). Under these conditions, BestKeeper ranked RNU48 as the best reference gene, followed by miR-874, miR-151-5p, and Z30.

The comparison of the summarized data in Table 2 shows that the results provided by NormFinder and BestKeeper displayed slight differences from the geNorm analysis but did have some overlap. While geNorm recommended miR-101, miR-125a-5p, miR-148b, and miR-151-5p for proper normalization, NormFinder indicated miR-148b as the best reference miRNA and miR-125a-5p as a part of the best combination. Additionally, BestKeeper identified miR-151-5p within the four most stably expressed miRNAs. As stated in the Introduction, the small nucleolar RNU6B is commonly used for miRNA expression normalization and in our study was ranked 10^th^ by geNorm, 12^th^ by NormFinder, and 9^th^ by BestKeeper (Table 2). Thus, RNU6B seems to be a rather inappropriate reference gene for the miRNA expression normalization in studies on bladder cancer.

### Influence of Reference miRNA Selection on the Accuracy of Relative Quantification

**Figure 3:**
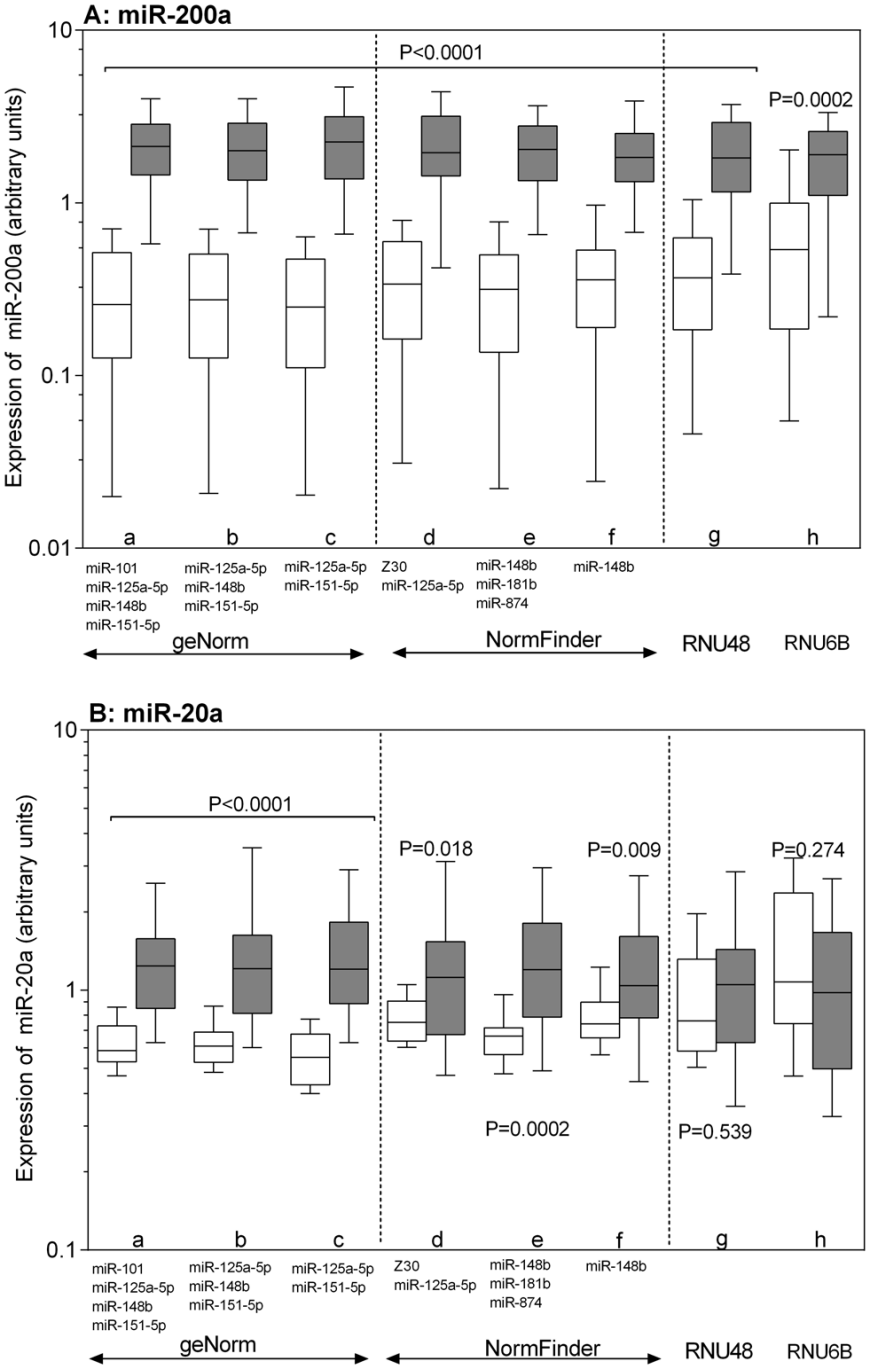
Effects of different normalization approaches on the expression of miR-200a and miR-20a. The relative expression of (A) miR-200a and (B) miR-20a as highly and moderately differentially expressed miRNAs, respectively was calculated using the following normalization strategies recommended by geNorm (a–c), NormFinder (d–f), BestKeeper (g), and RNU6B (h). The geNorm approaches were: (a) the four-reference-miRNA combination recommended as necessary number of reference miRNAs (miR-101, miR-125a-5p, miR-148b, miR-151-5p); (b) the three best ranked miRNAs according to their M values (miR-125a-5p, miR-148b, and miR-151-5p) and (c) the best two-gene-reference combination (miR-125a-5p, miR-151-5p). NormFinder normalization approaches were: (d) the best two reference gene combination (miR-125a-5p, Z30); (e) the three best ranked reference genes (miR-148b, miR-181b, miR-874); (f) the best single miRNA, miR-148b. BestKeeper normalization approach was (g) RNU48; (j) RNU6B as the most frequently recommended normalizer in bladder cancer studies. Values are given as arbitrary units; boxes (blank, nonmalignant tissue; black, malignant tissue) represent lower and upper quartiles with medians as horizontal line; whiskers depict the 10–90 percentiles. Significances are illustrated as *P* values of the Mann-Whitney *U* test.

To illustrate the impact of reference gene selection on miRNA expression analysis, we applied different normalization strategies for the relative quantification of miR-200a and miR-20a (Figure 3A–B). A preliminary evaluation of the miRNA microarray data showed a strong up-regulation of miR-200a (fold change 22.1) and a less robust, but significant up-regulation of miR-20a (fold change 2.78) in the tumor samples compared to the nonmalignant tissue samples. Different normalization approaches were used based on the recommendations by geNorm, NormFinder, and BestKeeper as described above. As shown in Figure 3, we normalized the expression of miR-200a and miR-20a using the geNorm recommended reference miRNAs as follows: (a) the combination of four reference miRNAs that were suggested to be necessary for reliable normalization (geometric mean of miR-101, miR-125a-5p, miR-148b, and miR-151-5p; Table 2); (b) the three best ranked miRNAs according to their M values (miR-125a-5p, miR-148b, and miR-151-5p), and (c) the best combination of two miRNAs (miR-125a-5p and miR-151-5p). The NormFinder recommended approaches were the following: (d) the best two reference gene combination (miR-125a-5p and Z30); (e) the three best ranked reference miRNAs (miR-148b, miR-181b, and miR-874), and (f) the best single miRNA miR-148b. Based on the BestKeeper recommendation, we also used (g) the calculated best single reference gene RNU48. In addition, we performed normalization with (h) RNU6B, which was estimated by all three programs to have low usefulness as a reference gene but is frequently used in expression studies. Regardless of the normalization approach, miR-200a was found to be up-regulated (Figure 3A). However, the expression pattern of miR-20a was different depending on the normalization approach (Figure 3B). Using the reference miRNAs recommended by geNorm or NormFinder, miR-20a appeared to be up-regulated in tumor samples, whereas normalization with RNU6B or RNU48 as recommended by BestKeeper did not show up-regulation of this miRNA. Thus, although all reference miRNA suggestions by geNorm and NormFinder were obviously suited to be appropriate for normalization, we recommend the use of more than two reference miRNAs preferring the use of four miRNAs (miR-101, miR-148b, miR-125a-5p, and miR-151-5p) as recommended by geNorm or the combination of three miRNAs (miR-148b, miR-181b, and miR-874) suggested by NormFinder. The two-miRNA combinations or single miRNAs should be cautiously considered as alternative normalization approaches only if limited sample material is available for analysis.

## Discussion

The selection of suitable reference genes as normalizers for relative quantification of mRNA and miRNA expression is essential to avoid erroneous expression results and to improve the comparability of gene expression data between different studies. Different models such as the global mean normalization [5], panels of miRNAs [37] or small RNAs [53] have been suggested for the normalization of miRNA expression data. D’haene et al. [54] recently reported that the side-by-side comparison of small nuclear RNA normalization with global mean normalization indicated that small nuclear RNAs are less efficient in reducing the technical variation and do not reveal in accurate expression differences. In addition, the recommended global mean normalization method [5] that is also included in the algorithm of the qBase^PLUS^ software requires a large number of genes, for example in microarray, deep sequencing, bead-based or TaqMan array card analyses. Thus, the global normalization approach is not feasible in RT-qPCR studies because only a few miRNAs are generally measured. In this case, the normalization of miRNA RT-qPCR data with suitable miRNA reference markers can be considered as the method of choice [55].

Studies to identify and validate suitable reference miRNAs have been performed for several cancers [13], [37]–[40]. As discussed in the Introduction, it is therefore quite astonishing that no miRNA expression studies in bladder cancer have used endogenous miRNAs for normalization. Only nuclear, nucleolar, and ribosomal RNAs as well as mRNAs have been used. However, the different lengths of these RNAs compared to miRNAs result in different physico-chemical properties with different isolation efficiencies and degradation [56], [57]. miRNAs are more stable than mRNAs or small RNAs like RNU6B, and they can therefore be more accurately detected in tissues [57]. In addition, different techniques of reverse transcription used for miRNAs and the other RNAs make the latter less suitable for normalization. Furthermore, as previously shown for mRNAs, the tissue-specific expression of miRNAs is also reflected in the behavior of putative endogenous reference genes [14], [15]. Thus, RT-qPCR-based miRNA expression studies optimally require normalization by reference miRNAs. Previous reports from our group have demonstrated the importance of suitable reference miRNAs in avoiding biased results in miRNA expression studies in other urological tumors [13], [39]. These data strongly support the need for determining appropriate endogenous reference miRNAs to allow stringent normalization of miRNA expression patterns in urothelial carcinoma.

To the best of our knowledge, the present study is the first systematic investigation of suitable normalizers for relative quantification of miRNA expression in bladder cancer. In this study, we combined different strategies for identifying suitable reference miRNAs. A four step approach was used. First, to obtain an overview of the miRNAome in bladder cancer tissue, miRNA microarray analyses for nonmalignant and malignant bladder samples were performed to identify invariant miRNAs as stably-expressed candidate reference miRNAs within the data set. Second, these candidate reference miRNAs were validated by RT-qPCR, in addition to RNU6B, RNU48, and Z30, the most frequently reported reference genes for miRNA expression studies in bladder cancer. Third, the statistical algorithms geNorm, NormFinder, and BestKeeper were applied to identify the most useful endogenous reference miRNAs for relative quantification. Finally, the impact of different normalization approaches was illustrated for two deregulated miRNAs in bladder cancer tissue to emphasize the importance of an appropriate normalization approach.

The starting point of the present study was miRNA microarray analysis. According to the criteria for the microarray data evaluation and the measurability criterion for subsequent RT-qPCR analysis (Cq values <35), 11 invariant miRNAs were identified to be putative reference miRNAs. Since miR-151-3p and miR-151-5p derive from the same pre-miRNA and miR-151-5p exhibited slightly higher expression in examined samples, we included only miR-151-5p in further analysis. A data search in the miRNA bladder cancer studies mentioned in the Introduction showed that miR-29c, one of these 11 invariant, stably expressed miRNAs, was found to be down-regulated in two microarray studies [19], [36]. Our subsequent RT-qPCR confirmed this observation (Figure 1). Although we did not eliminate this miRNA from the subsequent analysis for the validation of suitable reference miRNAs through geNorm, NormFinder, and BestKeeper, miR-29c was never recommended as a reference miRNA by one of these evaluation tools in our following analyses. This finding also indicates the usefulness of the software packages in the search for suitable reference genes.

The putative reference miRNAs identified by the microarray analyses, except miR-151-3p as mentioned, were included with the additional RNAs RNU6B, RNU48, and Z30 in the geNorm, NormFinder, and BestKeeper analysis. Differences in expression observed in the subsequent RT-qPCR measurements between nonmalignant, low-grade, and high-grade tumor samples as well as co-expressions of genes did not exclude candidate reference genes. However, as comprehensively described in the Results section, geNorm, NormFinder, and BestKeeper did not always recommend the same reference miRNAs for normalization (Table 2). The lack of agreement between geNorm and NormFinder results has been described previously [15]. The reasons for these differences in the ranking order of the putative reference miRNAs might be due to the different calculation models on which the tools are based. NormFinder is an ANOVA-based model, geNorm uses a pairwise comparison model, and BestKeeper determines the optimal reference genes by employing the pairwise correlation analysis on all pairs of candidate reference genes. While the geNorm approach is theoretically robust with regard to inter-sample variations arising from sources such as differing RNA input and quality, it has been shown to prefer co-regulated genes in the selection as normalizers [10]. In this study, geNorm also recommended co-regulated reference miRNAs (miR-101 with miR-125a-5p, miR-151-5p) but miR-324-3p was never recommended as normalizer despite its strong correlation with miR-101 and miR-151-5p.

The differences between the recommended reference miRNAs by the three programs prompted us to validate their suitability in clinical samples (Figure 3A–B). The importance of selecting suitable reference genes for accurate miRNA expression data has been shown not only in mRNA but also in miRNA expression studies [13], [37], [39], [40]. We tested the suitability of the different approaches with miR-200a, a highly up-regulated miRNA, and miR-20a, which is up-regulated less robustly (Figure 3A, B). The results clearly demonstrated that RNUB6, which is the most frequent normalizer used in previous miRNA expression studies in bladder cancer, and RNU48, which was recommended by BestKeeper, were unable to confirm the small expression changes, e.g. for miR-20a. The poor quality of RNU6B as a reference gene has already been reported in miRNA expression studies in renal cell carcinoma and prostate cancer [13], [58], where its altered expression stability depended on the degradation of the RNA as compared with miRNAs [13]. In contrast, all geNorm and NormFinder recommendations for single and multiple reference miRNA combinations proved to be suitable normalization approaches in the present study for revealing not only strongly but also less robustly deregulated miRNA expression levels between nonmalignant and malignant urothelial tumor samples. However, we recommend the combination of four (miR-101, miR-125a-5p, miR-148b, and miR-151-5p) or three (miR-148b, miR-181b, and miR-874) reference miRNAs. Although the normalization with the best single (NormFinder) or the best two (geNorm) reference miRNAs in our study gave comparable results to the larger gene sets, the use of multiple reference miRNAs is critical in achieving more reliable expression data [7]–[10].

In summary, the present study was the first systematic investigation to identify suitable reference miRNAs in a transparent and comprehensive manner for the relative quantification of the microRNAome in urothelial carcinoma. It was based on a four-step approach with microarray analyses, RT-qPCR validation, reference miRNA selection through computer software, and proof of principle with different miRNA expression levels. Starting with 16 putative reference miRNAs from the microarray analysis and three additional small RNAs from the literature, we validated several combinations of reference miRNAs for miRNA expression studies in bladder cancer. We believe that these are robust methods that will allow future studies on the functional roles of miRNAs as regulators in signal transduction and metabolic pathways that are associated with small expression changes.

## Supporting Information

Table S1 **Description of the experimental details of the RT-qPCR analyses according to the checklist of the MIQE guidelines.**
(PDF)

Table S2 **TaqMan assays for microRNAs and small nuclear and nucleolar RNAs.**
(PDF)

Table S3 **List of the 101 miRNAs from the microarray for the identification of candidate reference miRNAs.**
(PDF)

Table S4 **Spearman rank correlation coefficients (r_s_) between the candidate reference genes.**
(PDF)

Methods S1 **qPCR validation experiments according to the MIQE guidelines with respect to the calibration curves and the dynamic range of measurements.**
(PDF)
