# Supplementary material for: Reference miRNAs for miRNAome Analysis of Urothelial Carcinomas
Source: PLoS One. 2012 Jun 20;7(6):e39309. doi: 10.1371/journal.pone.0039309 (PMC3380005; doi:10.1371/journal.pone.0039309)
Supplement: Table S1 — Description of the experimental details of the RT-qPCR analyses according to the checklist of the MIQE guidelines. (PDF) [file pone.0039309.s001.pdf]

Table S1 Description of the experimental details of the RT-qPCR analyses according to the checklist of the MIQE guidelines.

The items in the checklist summarize the characteristics of the RT-qPCR analyses according to Bustin et al. [Clin Chem (2009) 611-622] and indicate where the details are described in the manuscript.

ITEM TO CHECK	IMPOR-TANCE[&]	CHECK-LIST	WHERE IN THE MANUSCRIPT; ADDITIONAL COMMENT
**ERXPERIMENTAL DESIGN**			
Definition of experimental and control groups	**E**	Yes	Materials and Methods: Patients and tissue samples.
Number within each group	**E**	Yes	Materials and methods: Patients and tissue samples.
Assay carried out by core lab or investigator´s lab?	D	Yes	All assays were performed in investigator´s lab.
Acknowledgement of authors´ contributions	D	Yes	Contributors who do not meet the authorship as defined by the journal are listed in the Acknowledgement section.
**SAMPLE**			
Description	**E**	Yes	Materials and Methods.
Volume/mass of sample processed	**D**	Yes	Materials and Methods: Isolation of RNA....
Microdissection or macrodissection	**E**	Yes	Materials and Methods: Isolation of RNA....; macrodissections with histological verification.
Processing procedure	**E**	Yes	Materials and Methods: Patients and tissue samples; Isolation of RNA...
If frozen – how and how quickly?	**E**	Yes	Materials and Methods: Patients and tissue samples.
If fixed – with what, how quickly?	**E**	N/A	-
Samples storage conditions and duration (esp. for FFPE samples)	**E**	Yes	Materials and Methods: Patients and tissue samples.
**NUCLEIC ACID EXTRACTION**			
Procedure and/or instrumentation	**E**	Yes	Materials and Methods: Isolation of RNA and characterization of quantity and quality; references are indicated.
Name of kit and details of any modifications	**E**	Yes	Materials and Methods: Isolation of RNA and characterization of quantity and quality; references are indicated.
Source of additional reagents used	**E**	Yes	RNase-free DNase set; Qiagen (cat.no. 79254); see subsequent information.
Details of DNase or RNase treatment	**E**	Yes	Materials and Methods: Isolation of RNA....; an optional on column digestion DNase step was used.
Contamination assessment (DNA or RNA)	**E**	Yes	See previous comment; according to Chen et al. (Nucleic Acids Res 33(2005) e179) miRNA measurements by the TaqMan assays are not affected by genomic DNA; see also comment on Cqs with and without RT.
Nucleic acid quantification	**E**	Yes	Materials and Methods: Isolation of RNA and characterization of quantity and quality; references are indicated.
Instrument and method	**E**	Yes	Materials and Methods: Isolation of RNA and characterization of quantity and quality; references are indicated.
Purity (A260/A280)	D	Yes	Materials and Methods: Isolation of RNA and characterization of quantity and quality; references are indicated.
Yield	D	Yes	Materials and Methods: Isolation of RNA and characterization of quantity and quality.
RNA integrity method/instrument	**E**	Yes	Materials and Methods: Isolation of RNA and characterization of quantity and quality; Bioanalyzer 2100 (Agilent).
RIN/RQI or Cq of 3`and 5`transcripts	**E**	Yes	RIN values; given in Materials and Methods: Isolation of RNA and characterization of quantity and quality.
Electrophresis traces	D	No	-
Inhibition testing (Cq dilutions, spike or other)	**E**	Yes	Dilution experiments were performed; PCR efficiencies were found 81%-88%; see also Supporting Information, Methods S1. For all clinical samples, identical isolation procedures were performed.
**REVERSE TRANSCRIPTION**			
Complete reaction condition	**E**	Yes	Materials and Methods: Quantitative real-time PCR; references are indicated.
Amount of RNA and reaction volume	**E**	Yes	Materials and Methods: Quantitative real-time PCR; references are indicated.

ITEM TO CHECK	IMPOR-TANCE[&]	CHECK-LIST	WHERE IN THE MANUSCRIPT; ADDITIONAL COMMENT
Priming oligonucleotide (if using GSP) and concentration	E	Yes	Materials and Methods: Quantitative real-time PCR; references are indicated.
Reverse transcriptase and concentration	E	Yes	Materials and Methods: Quantitative real-time PCR; references are indicated.
Temperature and time	E	Yes	Materials and Methods: Quantitative real-time PCR; references are indicated.
Manufacturer and reagents and catalogue numbers	D	Yes	Materials and Methods: Quantitative real-time PCR; references are indicated.; see also Supporting Information, Table S2.
Cqs with and without RT	D*	Yes	There were no Cqs < 40 in reactions without RT.
Storage conditions of cDNA	D	Yes	Materials and Methods: Quantitative real-time PCR; -20°C.
**qPCR TARGET INFORMATION**			
If multiplex, efficiency and LOD of each assay	E	N/A	-
Sequence accession number	E	Yes	Table 1 and Supporting Information, Table S2.
Location of amplicon	D	Yes	Use of miRNAs specific TaqMan assays; specificity guaranteed by the manufacturer.
Amplicon length	E	N/A	Use of miRNAs specific TaqMan assays; specificity guaranteed by the manufacturer.
In silico specificity screen (BLAST, etc.)	E	N/A	Use of miRNA specific TaqMan assays; specificity guaranteed by the manufacturer.
Pseudogenes, retropseudogenes or other homologs?	D	N/A	Use of miRNA specific TaqMan assays; specificity guaranteed by the manufacturer.
Sequence alignment	D	N/A	Use of miRNA specific TaqMan assays; specificity guaranteed by the manufacturer.
Secondary structure analysis of amplicon	D	N/A	Use of miRNA specific TaqMan assays; specificity guaranteed by the manufacturer.
Location of each primer by exon or intron (if applicable)	E	Yes	Use of miRNA specific TaqMan assays; specificity guaranteed by the manufacturer.
What splice variants are targeted?	E	Yes	Specificity guaranteed by manufacturer of the TaqMan assays.
**qPCR OLIGONUCLEOTIDES**			
Primer sequences	E	N/A	The manufacturer does not provide this information for miRNAs; see also Supporting Information, Table S2.
RTPrimerDB Identification Number	D	N/A	Use of miRNA specific TaqMan assays; see also Supporting Information, Table S2.
Probe sequences	D**	N/A	The manufacturer does not provide this information for miRNAs; see also Supporting Information, Table S2.
Location and identity of any modifications	E	Yes	The manufacturer does not provide this information for miRNAs; see also Supporting Information, Table S2.
Manufacture of oligonucleotides	D	Yes	Applied Biosystems as part of Life Technologies.
Purification method	D	No	Applied Biosystems does not provide information.
**qPCR PROTOCOL**			
Complete reaction conditions	**E**	Yes	Materials and Methods: Quantitative real-time PCR; references are indicated; use of miRNA specific TaqMan assays.
Reaction volume and amount of cDNA/DNA	E	Yes	Materials and Methods: Quantitative real-time PCR; references are indicated.
Primer, (probe), Mg++ and dNTP concentration	E	Yes	Materials and Methods: Quantitative real-time PCR; references are indicated; use of miRNA specific TaqMan assays.
Polymerase identity and concentration	E	Yes	Materials and Methods: Quantitative real-time PCR; references are indicated; use of miRNA specific TaqMan assays.
Buffer/kit identity and manufacture	E	No	Materials and Methods: Quantitative real-time PCR; references are indicated; use of miRNA specific TaqMan assays.
Exact chemical constitution of the buffer	D	Yes	The manufacturer does not provide this information.
Additives (SYBR Green I, DMSO, ect.)	E	Yes	Use of TaqMan assays without additional additives.
Manufacturer of plates/tubes and catalog number	D	Yes	Materials and Methods: Quantitative real-time PCR; white 96-well PCR plates (Roche; cat.no. 04729692001 with sealing foils)
Complete thermocylingparameter	E	Yes	Materials and Methods: Quantitative real-time PCR.
Reaction setup (manual/robotic)	D	Yes	Manual setup.
Manufacturer of qPCR instruments	E	Yes	LightCycler 480; see also Materials and Methods: Quantitative real-time PCR.
**qPCR VALIDATION**			
Evidence of optimmisation (from gradients)	D	Yes	Kits from Applied Biosystems; optimization guaranteed by the manufacturer.
Specificity (gel, sequence, melt, or digest)	E	Yes	Specificity guaranteed by manufacturer of the TaqMan assays.

ITEM TO CHECK	IMPOR-TANCE&	CHECK-LIST	WHERE IN THE MANUSCRIPT; ADDITIONAL COMMENT
For SYBR Green I, Cq of the NTC	E	Not applicable	-
Calibration curves with slope and Y-intercept	E	Yes	Material and Methods: Quantitative real-time PCR; see also Supporting Information, Methods S1.
PCR efficiency calculated from slope	E	Yes	Material and Methods: Quantitative real-time PCR; see also Supporting Information, Methods S1.
Confidence interval PCR efficiency or standard error	D	Yes	Supporting Information, Methods S1.
r2 of standard curve	E	No	Not provided by the LightCycler 480 software.
Linear dynamic range	E	Yes	Material and Methods: Quantitative real-time PCR; see also Supporting Information, Methods S1.
Cq variation at lowest concentration of the linear interval of calibration curves	E	Yes	Supporting Information, Methods S1.
Confidence intervals throughout range	D	No	-
Evidence for limit of detection	E	Yes	Material and Methods: Quantitative real-time PCR; see also Supporting Information, Methods S1. Measurements of all miRNAs were in linear dynamic range. Thus, it was not necessary to determine the LOD.
If multiplex, efficiency and LOD of each assay	E	N/A	-
**DATA ANALYSIS**			
qPCR analysis program (source, version)	E	Yes	Materials and Methods: Quantitative real-time PCR and Supporting Information, Methods S1.
Cq method determination	E	Yes	Cq >35 was decided as limit.
Outlier identification and disposition	E	Yes	There were no outliers.
Results of NTCs	E	Yes	NTC did not result in any amplification; Cq >40.
Justification of number and choice of reference genes	E	Yes	See Results/Discussion.
Description of normalization method	E	Yes	Materials and Methods: Data analysis, use of geNorm, NormFinder, BestKeeper.
Number and concordance of biological replicates	D	Yes	Figure 1 and 3: nonmalignant n = 17; low-grade tumor samples n = 20; high-grade tumor samples n= 21.
Number and stage (RT or qPCR) of technical replicates	E	Yes	Materials and Methods: Quantitative real-time PCR; triplicate measurements.
Repeatability (intra-assay variation, %CV)	E	Yes	Materials and Methods: Quantitative real-time PCR
Reproducibility (inter-assay variation, %CV)	D	Yes	Materials and Methods: Quantitative real-time PCR; in addition; biological replicates were preferred in favor of technical replicates.
Power analysis	D	No	-
Statistical methods for result significance	E	Yes	Materials and Methods: Data analysis; Results and Figure legends.
Software (source, version)	E	Yes	Materials and Methods: Data analysis.
Cq or raw data submission RDML	D	No	-

&E, essential information that must be submitted with the manuscript; D, desirable information that should be submitted with the manuscript if available.
