# Supplementary material for: Reference miRNAs for miRNAome Analysis of Urothelial Carcinomas
Source: PLoS One. 2012 Jun 20;7(6):e39309. doi: 10.1371/journal.pone.0039309 (PMC3380005; doi:10.1371/journal.pone.0039309)
Supplement: Table S2 — TaqMan assays for microRNAs and small nuclear and nucleolar RNAs. (PDF) [file pone.0039309.s002.pdf]

Table S2 TaqMan assays for microRNAs and small nuclear and nucleolar RNAs.

TaqMan assays from Applied Biosystems for the examined mature miRNAs and small nuclear RNAs are listed. The assay name, assay ID, miRBase accession number, miRBase ID, and the sequence are given for the miRNAs and correspondingly for the small nuclear and nucleolar RNAs. miRNAs were identified by the permanently assigned miRBase accession number, the miRBase-prescribed ID related to the miRBase version, and the sequence.

Assay name	Assay ID	miRBase accession no.	miRBase ID[&]	Sequence
hsa-miR-15a	000389	MIMAT0000068	hsa-miR-15a (v10.1)<br>hsa-miR-15a-5p (v18)	UAGCAGCACAUAAUGGUUUGUG
hsa-miR-20b	000580	MIMAT0001413	hsa-miR-20b (v10.1)<br>hsa-miR-20b-5p (v18)	CAAAGUGCUCAUAGUGCAGGUAG
hsa-miR-29c	000587	MIMAT0000681	hsa-miR-29c (v10.1)<br>hsa-miR-29c-3p(v18)	UAGCACCAUUUGAAAUCGGUUA
hsa-miR-101	002253	MIMAT0000099	hsa-miR-101 (v10.1)<br>hsa-miR-101-3p (v18)	UACAGUACUGUGAUAACUGAA
hsa-miR-107	000443	MIMAT0000104	hsa-miR-107 (v10.1)<br>hsa-miR-107 (v18)	AGCAGCAUUGUACAGGGCUAUCA
hsa-miR-125a-5p	002198	MIMAT0000443	hsa-miR-125a-5p (v10.1)<br>hsa-miR-125a-5p (v18)	UCCCUGAGACCCUUUAACCUGUGA
hsa-miR-148b	000417	MIMAT0000759	hsa-miR-148b (v10.1)<br>hsa-miR-148b-3p (v18)	UCAGUGCAUCACAGAACUUUGU
hsa-miR-151-3p	002254	MIMAT0000757	hsa-miR-151-3p (v10.1)<br>hsa-miR-151-3p (v18)	CUAGACUGAAGCUCCUUGAGG
hsa-miR-151-5p	002642	MIMAT0004697	hsa-miR-151-5p (v10.1)<br>hsa-miR-151-5p (v18)	UCGAGGAGCUCACAGUCUAGU
hsa-miR-181a	000480	MIMAT0000256	hsa-miR-181a (v10.1)<br>hsa-miR-181a-5p (v18)	AACAUUCAACGCUGUCGGUGAGU
hsa-miR-181b	001098	MIMAT0000257	hsa-miR-181b (v10.1)<br>hsa-miR-181b-5p (v18)	AACAUUCAUUGCUGUCGGUGGGU
hsa-miR-324-3p	002161	MIMAT0000762	hsa-miR-324-3p (v10.1)<br>hsa-miR-324-3p (v18)	ACUGCCCCAGGUGCUGCUGG

**Assay name**	**Assay ID**	**miRBase accession no.**	**miRBase ID[&]**	**Sequence**
hsa-miR-424	000604	MIMAT0001341	hsa-miR-424 (v10.1)<br>hsa-miR-424-5p (v18)	CAGCAGCAAUUCAUGUUUUGAA
hsa-miR-513a-5p	002090	MIMAT0002877	hsa-miR-513a-5p (v10.1)<br>hsa-miR-513a-5p (v18)	UUCACAGGGAGGUGUCAU
hsa-miR-874	002268	MIMAT0004911	hsa-miR-874 (v10.1)<br>hsa-miR-874 (v18)	CUGCCCUGGCCCGAGGGACCGA
hsa-miR-939	002182	MIMAT0004982	hsa-miR-939 (v10.1)<br>hsa-miR-939 (v18)	UGGGGAGCUGAGGCUCUGGGGGUG

[&]miRNA ID in the miRBase version 10.1 and 18, respectively.

**Gene name**	**Assay ID**	**Assay name**	**Sequence**
RNU6B	001093	568915	CGCAAGGATGACACGCAAATTCGTGAAGCGTTCCATATTTTT
RNU48	001006	568908	GATGACCCCAGGTAACTCTGAGTGTGTCGCTGATGCCATCACCGCAGCGCTCTGACC
Z30	001092	568917	TGGTATTGCCATTGCTTCACTGTTGGCTTTGACCAGGGTATGATCTCTTAATCTTCTCTCTGAGCTG
