# Supplementary material for: Reference miRNAs for miRNAome Analysis of Urothelial Carcinomas
Source: PLoS One. 2012 Jun 20;7(6):e39309. doi: 10.1371/journal.pone.0039309 (PMC3380005; doi:10.1371/journal.pone.0039309)
Supplement: Table S3 — List of the 101 miRNAs from the microarray for the identification of candidate reference miRNAs. (PDF) [file pone.0039309.s003.pdf]

Table S3 List of the 101 miRNAs from the microarray for the identification of candidate reference miRNAs.

All miRNAs of the microarray are listed that were flagged as "present" in all examined 24 tissue samples by the Genespring GX11 software.

	miRNA	Active sequence on the microarray
**1**	**hsa-let-7a**	AACTATACAACCTACTACCT
**2**	**hsa-let-7b**	AACCACACAACCTACTACC
**3**	**hsa-let-7c**	AACCATACAACCTACTACC
**4**	**hsa-let-7d**	AACTATGCAACCTACTACC
**5**	**hsa-let-7e**	AACTATACAACCTCCTACC
**6**	**hsa-let-7f**	AACTATACAATCTACTACCTC
**7**	**hsa-let-7g**	AACTGTACAAACTACTACCTC
**8**	**hsa-let-7i**	AACAGCACAAACTACTACCTC
**9**	**hsa-miR-10b**	CACAAATTCGGTTCTACAGGG
**10**	**hsa-miR-15a**	CACAAACCATTATGTGCTGCT
**11**	**hsa-miR-15b**	TGTAAACCATGATGTGCTGC
**12**	**hsa-miR-16**	CGCCAATATTTACGTGCTG
**13**	**hsa-miR-17**	CTACCTGCACTGTAAGC
**14**	**hsa-miR-19a**	TCAGTTTTGCATAGATTTGCA
**15**	**hsa-miR-19b**	TCAGTTTTGCATGGATTTGC
**16**	**hsa-miR-20a**	CTACCTGCACTATAAGCAC
**17**	**hsa-miR-20b**	CTACCTGCACTATGAGCAC
**18**	**hsa-miR-21**	TCAACATCAGTCTGATAAGC
**19**	**hsa-miR-22**	ACAGTTCTTCAACTGGCAG
**20**	**hsa-miR-23a**	GGAAATCCCTGGCAATGT
**21**	**hsa-miR-23b**	GGTAATCCCTGGCAATG
**22**	**hsa-miR-24**	CTGTTCCTGCTGAACTGA
**23**	**hsa-miR-25**	TCAGACCGAGACAAGTGC
**24**	**hsa-miR-26a**	AGCCTATCCTGGATT
**25**	**hsa-miR-26b**	ACCTATCCTGAATTACTTGA
**26**	**hsa-miR-27a**	GCGGAACTTAGCCACTG
**27**	**hsa-miR-27b**	GCAGAACTTAGCCACTGT
**28**	**hsa-miR-28-5p**	CTCAATAGACTGTGAGCTCC
**29**	**hsa-miR-29a**	TAACCGATTTCAGATGGTGC
**30**	**hsa-miR-29b**	AACACTGATTTCAAATGGTGC
**31**	**hsa-miR-29c**	TAACCGATTTCAAATGGTGCTA
**32**	**hsa-miR-29c***	GAACACCAGGAGAAATCGGT
**33**	**hsa-miR-30a**	CTTCCAGTCGAGGATG
**34**	**hsa-miR-30b**	AGCTGAGTGTAGGATGTT
**35**	**hsa-miR-30c**	GCTGAGAGTGTAGGATGT
**36**	**hsa-miR-30d**	CTTCCAGTCGGGGA
**37**	**hsa-miR-30e**	CTTCCAGTCAAGGATGT
**38**	**hsa-miR-34a**	ACAACCAGCTAAGACACTGC
**39**	**hsa-miR-92a**	ACAGGCCGGGACAAGT
**40**	**hsa-miR-93**	CTACCTGCACGAACAG
**41**	**hsa-miR-99b**	CGCAAGGTCGGTTCTA
**42**	**hsa-miR-101**	TTCAGTTATCACAGTACTGT
**43**	**hsa-miR-103**	TCATAGCCCTGTACAATG
**44**	**hsa-miR-106b**	ATCTGCACTGTCAGCAC

	miRNA	Active sequence on the microarray
**45**	**hsa-miR-107**	TGATAGCCCTGTACAATGCT
**46**	**hsa-miR-125a-3p**	GGCTCCCAAGAACCTCA
**47**	**hsa-miR-125a-5p**	TCACAGGTTAAAGGGTCTC
**48**	**hsa-miR-125b**	TCACAAGTTAGGGTCTC
**49**	**hsa-miR-126**	CGCATTATTACTCACGGT
**50**	**hsa-miR-130a**	ATGCCCTTTTAACATTGCA
**51**	**hsa-miR-130b**	ATGCCCTTTCATCATTGC
**52**	**hsa-miR-140-3p**	CCGTGGTTCTACCCT
**53**	**hsa-miR-140-5p**	CTACCATAGGGTAAAACCACT
**54**	**hsa-miR-141**	CCATCTTTACCAGACAG
**55**	**hsa-miR-142-3p**	TCCATAAAGTAGGAAACACTACA
**56**	**hsa-miR-143**	GAGCTACAGTGCTTC
**57**	**hsa-miR-145**	AGGGATTCCTGGGAAAAC
**58**	**hsa-miR-148a**	ACAAAGTTCTGTAGTGCACT
**59**	**hsa-miR-148b**	ACAAAGTTCTGTGATGCAC
**60**	**hsa-miR-151-3p**	CCTCAAGGAGCTTCAGT
**61**	**hsa-miR-151-5p**	ACTAGACTGTGAGCTCC
**62**	**hsa-miR-181a**	ACTCACCGACAGCGT
**63**	**hsa-miR-181b**	ACCCACCGACAGCA
**64**	**hsa-miR-185**	TCAGGAACTGCCTTTCT
**65**	**hsa-miR-193b**	AGCGGGACTTTGAGGG
**66**	**hsa-miR-195**	GCCAATATTTCTGTGCTGC
**67**	**hsa-miR-199a-3p**	TAACCAATGTGCAGACTACT
**68**	**hsa-miR-199a-5p**	GAACAGGTAGTCTGAACAC
**69**	**hsa-miR-200a**	ACATCGTTACCAGACAGT
**70**	**hsa-miR-200b**	TCATCATTACCAGGCAG
**71**	**hsa-miR-200c**	TCCATCATTACCCGG
**72**	**hsa-miR-205**	CAGACTCCGGTGGAAT
**73**	**hsa-miR-210**	TCAGCCGCTGTCACAC
**74**	**hsa-miR-223**	TGGGGTATTTGACAAACTGAC
**75**	**hsa-miR-320a**	TCGCCCTCTCAAC
**76**	**hsa-miR-324-3p**	CCAGCAGCACCTGGGG
**77**	**hsa-miR-331-3p**	TTCTAGGATAGGCCCAGGG
**78**	**hsa-miR-342-3p**	ACGGGTGCGATTTCTG
**79**	**hsa-miR-361-5p**	GTACCCCTGGAGATTC
**80**	**hsa-miR-365**	ATAAGGATTTTTAGGGGCATTA
**81**	**hsa-miR-374a**	CACTTATCAGGTTGTATTATAA
**82**	**hsa-miR-378**	CCTTCTGACTCCA
**83**	**hsa-miR-423-5p**	AAAGTCTCGCTCTCTG
**84**	**hsa-miR-424**	TTCAAAACATGAATTGCTGCTG
**85**	**hsa-miR-425**	TCAACGGGAGTGATCGTG
**86**	**hsa-miR-429**	ACGGTTTTACCAGACAGTA
**87**	**hsa-miR-451**	AACTCAGTAATGGTAACGGTTT
**88**	**hsa-miR-483-5p**	CTCCCTTCTTTCCTC
**89**	**hsa-miR-494**	GAGGTTTCCCGTGTA
**90**	**hsa-miR-497**	ACAAACCACAGTGTGCTG
**91**	**hsa-miR-513a-5p**	ATGACACCTCCCTGTG
**92**	**hsa-miR-575**	GCTCCTGTCCAACTGGCT

	miRNA	Active sequence on the microarray
**93**	**hsa-miR-638**	AGGCCGCCACCCGC
**94**	**hsa-miR-768-3p_v11.0**	GTCAGCAGTTTGAGTGTCAG
**95**	**hsa-miR-768-5p_v11.0**	ATCACTCCGTACTTTCATC
**96**	**hsa-miR-801_v10.1**	GTCGATTCCGCACGC
**97**	**hsa-miR-874**	TCGGTCCCTCGGG
**98**	**hsa-miR-886-3p**	AAGGGTCAGTAAGCACCCGC
**99**	**hsa-miR-923_v12.0**	AGTTTCTTTTCCTCCGC
**100**	**hsa-miR-939**	CACCCCCAGAGCC
**101**	**hsa-miR-1225-5p**	CCCCCCACTGGG
