# Supplementary material for: Reference miRNAs for miRNAome Analysis of Urothelial Carcinomas
Source: PLoS One. 2012 Jun 20;7(6):e39309. doi: 10.1371/journal.pone.0039309 (PMC3380005; doi:10.1371/journal.pone.0039309)
Supplement: Table S4 — Spearman rank correlation coefficients (rs) between the candidate reference genes. (PDF) [file pone.0039309.s004.pdf]

Table S4 Spearman rank correlation coefficients (r_s) between the candidate reference genes.[&]

miR		miR-29	miR-101	miR-125a-5p	miR-148b	miR-151-3p	miR-151-5p	miR-181a	miR-181b	miR-324-3p	miR-424	miR-874	RNU6B	RNU48	Z30
**miR-29c**	r_s		0.575	0.470	0.393	0.272	0.500	0.201	-0.163	0.386	0.264	0.583	0.143	0.557	0.174
	p-value		0.0001	0.0001	0.002	0.039	0.0001	0.131	0.221	0.003	0.045	0.0001	0.283	0.0001	0.192
**miR-101**	r_s	0.575		**0.621**	0.551	0.144	**0.622**	0.105	0.028	**0.630**	0.249	0.489	-0.074	0.196	0.257
	p-value	0.0001		0.0001	0.0001	0.280	0.0001	0.434	0.834	0.0001	0.059	0.0001	0.581	0.141	0.051
**miR-125a-5p**	r_s	0.470	**0.621**		0.380	0.266	**0.787**	0.314	0.150	**0.709**	0.306	0.341	-0.150	0.078	0.186
	p-value	0.0001	0.0001		0.003	0.044	0.0001	0.016	0.263	0.0001	0.020	0.009	0.261	0.560	0.161
**miR-148b**	r_s	0.393	0.551	0.380		**0.649**	0.530	0.357	0.220	0.440	0.315	0.368	0.137	0.381	0.524
	p-value	0.002	0.0001	0.003		0.0001	0.0001	0.006	0.096	0.001	0.016	0.004	0.306	0.003	0.0001
**miR-151-3p**	r_s	0.272	0.144	0.266	**0.649**		0.503	0.304	0.245	0.135	0.149	0.374	0.216	0.433	0.489
	p-value	0.039	0.280	0.044	0.0001		0.0001	0.021	0.064	0.313	0.266	0.004	0.104	0.001	0.0001
**miR-151-5p**	r_s	0.500	**0.622**	**0.787**	0.530	0.503		0.229	0.089	**0.672**	0.173	0.349	-0.115	0.239	0.298
	p-value	0.0001	0.0001	0.0001	0.0001	0.0001		0.084	0.505	0.0001	0.194	0.007	0.390	0.071	0.023
**miR-181a**	r_s	0.201	0.105	0.314	0.357	0.304	0.229		**0.746**	0.200	0.586	0.147	0.048	0.355	0.289
	p-value	0.131	0.434	0.016	0.006	0.021	0.084		0.0001	0.132	0.0001	0.272	0.722	0.006	0.028
**miR-181b**	r_s	-0.163	0.028	0.150	0.220	0.245	0.089	**0.746**		0.091	0.350	-0.046	0.053	0.052	0.231
	p-value	0.221	0.834	0.263	0.096	0.064	0.505	0.0001		0.498	0.007	0.733	0.694	0.698	0.081
**miR-324-3p**	r_s	0.386	**0.630**	**0.709**	0.440	0.135	**0.672**	0.200	0.091		0.214	0.224	-0.275	-0.045	0.092
	p-value	0.003	0.0001	0.0001	0.001	0.313	0.0001	0.132	0.498		0.107	0.090	0.037	0.736	0.491
**miR-424**	r_s	0.264	0.249	0.306	0.315	0.149	0.173	0.586	0.350	0.214		0.151	0.184	0.352	0.256
	p-value	0.045	0.059	0.020	0.016	0.266	0.194	0.0001	0.007	0.107		0.257	0.166	0.007	0.052
**miR-874**	r_s	0.583	0.489	0.341	0.368	0.374	0.349	0.147	-0.046	0.224	0.151		0.200	0.504	0.012
	p-value	0.0001	0.0001	0.009	0.004	0.004	0.007	0.272	0.733	0.090	0.257		0.132	0.0001	0.930
**RNU6B**	r_s	0.143	-0.074	-0.150	0.137	0.216	-0.115	0.048	0.053	-0.275	0.184	0.200		0.436	0.197
	p-value	0.283	0.581	0.261	0.306	0.104	0.390	0.722	0.694	0.037	0.166	0.132		0.001	0.139
**RNU48**	r_s	0.557	0.196	0.078	0.381	0.433	0.239	0.355	0.052	-0.045	0.352	0.504	0.436		0.353

	p-value	0.0001	0.141	0.560	0.003	0.001	0.071	0.006	0.698	0.736	0.007	0.0001	0.001		0.007
**Z30**	r_s	0.174	0.257	0.186	0.524	0.489	0.298	0.289	0.231	0.092	0.256	0.012	0.197	0.353	
	p-value	0.192	0.051	0.161	0.0001	0.0001	0.023	0.028	0.081	0.491	0.052	0.930	0.139	0.007	

[&]Correlations marked in yellow indicate r_S values ≥0.6.
