# Supplementary material for: Reference miRNAs for miRNAome Analysis of Urothelial Carcinomas
Source: PLoS One. 2012 Jun 20;7(6):e39309. doi: 10.1371/journal.pone.0039309 (PMC3380005; doi:10.1371/journal.pone.0039309)
Supplement: Methods S1 — qPCR validation experiments according to the MIQE guidelines with respect to the calibration curves and the dynamic range of measurements. (PDF) [file pone.0039309.s005.pdf]

Methods S1 qPCR validation experiments according to the MIQE guidelines with respect to the calibration curves and the dynamic range of measurements. Calibration curves were generated with diluted cDNAs (at least six dilution steps). The Cq values were calculated automatically by the LightCycler software, release 1.5.0 using the "second derivative maximum" method. The slopes, intercepts, and errors of the regression lines of the calibration curves from these dilution series and the PCR efficiencies ($E=10^{-1/slope}$) including the dynamic range and the Cq variation at the lower limit (the endpoint of the linear dynamic range) were also calculated by this software. Data and curves of the miR-26a, miR-151-3p, and RNU48 are exemplarily shown as follows. Since efficiencies did only differ in the second decimal place confirming the manufacturer's information that the TaqMan miRNA assays run with equivalent amplification efficiencies, we used the data of the calibration curve of miR-26a for all miRNAs and those of the curve of RNU48 for all RNUs.

**Gene**	**PCR-Efficiency**	**Slope**	**Y-Intercept**	**Error[&]**	**Linear dynamic range[#]**	**Cq variation at the lowest limit (SD)[§]**
miR-26a	1.862	-3.703	19.82	0.0459	19.02-35.29	0.17
miR-151-3p	1.884	-3.636	25.30	0.0653	24.31-35.60	0.11
RNU48	1.813	-3.870	22.22	0.0339	20.84-33.76	0.77

[&] The error value is the mean squared error of the single data points fit to the regression line, according to the definition given in the handbook of the LightCycler software.

[#] The linear dynamic range represents the range of the Cq values between the highest and the lowest concentration of linear interval of the calibration curve.

[§] Cq variation given as SD at the endpoint of the linear dynamic range that corresponds to the lowest concentration in the linear interval of the calibration curve.

Calibration curve of hsa-miR-26a

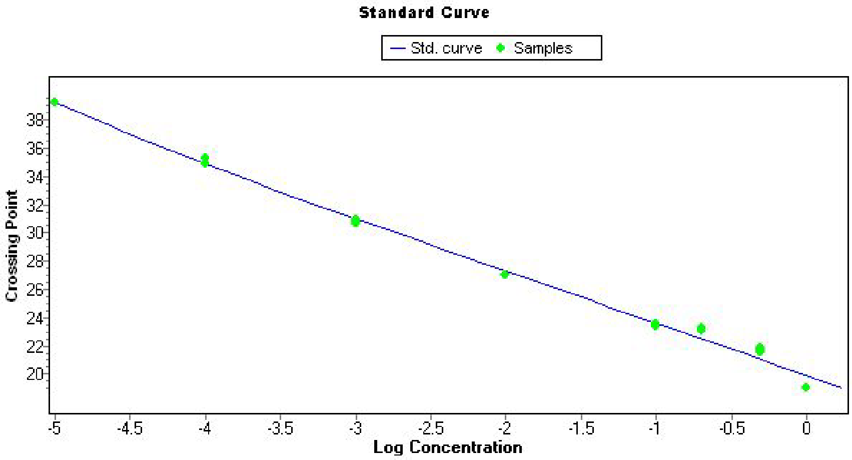

Error: 0.0459
Efficiency: 1.862
Slope: -3.703
Y-Intercept: 19.82

Calibration curve of hsa-miR-151-3p

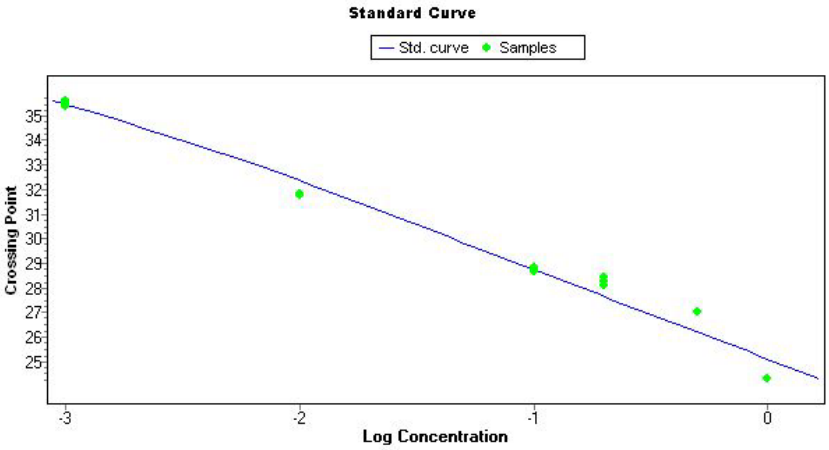

Error: 0.0653
Efficiency: 1.884
Slope: -3.636
Y-Intercept: 25.30

Calibration curve of RNU48

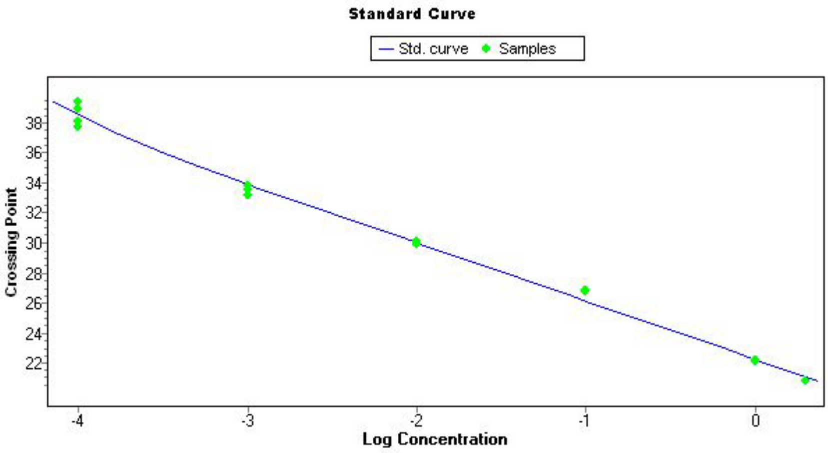

Error: 0.0339
Efficiency: 1.813
Slope: -3.870
Y-Intercept: 22.22
